# Analysis of Eukaryotic lincRNA Sequences Indicates Signatures of Hindered Translation Linked to Selection Pressure

**DOI:** 10.1093/molbev/msab356

**Published:** 2021-12-13

**Authors:** Anneke Brümmer, René Dreos, Ana Claudia Marques, Sven Bergmann

**Affiliations:** 1 Department of Computational Biology (DBC), University of Lausanne, Lausanne, Switzerland; 2 Swiss Institute of Bioinformatics (SIB), Lausanne, Switzerland; 3 Center for Integrative Genomics (CIG), University of Lausanne, Lausanne, Switzerland; 4 Department of Integrative Biomedical Sciences, University of Cape Town, Cape Town, South Africa

**Keywords:** noncoding RNA, computational sequence analysis, codon usage, tRNA abundance, ribosome binding, evolutionary selection pressure

## Abstract

Long intergenic noncoding RNAs (lincRNAs) represent a large fraction of transcribed loci in eukaryotic genomes. Although classified as noncoding, most lincRNAs contain open reading frames (ORFs), and it remains unclear why cytoplasmic lincRNAs are not or very inefficiently translated. Here, we analyzed signatures of hindered translation in lincRNA sequences from five eukaryotes, covering a range of natural selection pressures. In fission yeast and *Caenorhabditis elegans*, that is, species under strong selection, we detected significantly shorter ORFs, a suboptimal sequence context around start codons for translation initiation, and trinucleotides (“codons”) corresponding to less abundant tRNAs than for neutrally evolving control sequences, likely impeding translation elongation. For human, we detected signatures for cell-type-specific hindrance of lincRNA translation, in particular codons in abundant cytoplasmic lincRNAs corresponding to lower expressed tRNAs than control codons, in three out of five human cell lines. We verified that varying tRNA expression levels between cell lines are reflected in the amount of ribosomes bound to cytoplasmic lincRNAs in each cell line. We further propose that codons at ORF starts are particularly important for reducing ribosome-binding to cytoplasmic lincRNA ORFs. Altogether, our analyses indicate that in species under stronger selection lincRNAs evolved sequence features generally hindering translation and support cell-type-specific hindrance of translation efficiency in human lincRNAs. The sequence signatures we have identified may improve predicting peptide-coding and genuine noncoding lincRNAs in a cell type.

## Introduction

Long intergenic noncoding RNAs (lincRNAs) form a functionally heterogeneous class of RNAs longer than 200 nucleotides and lacking protein-coding potential (Ulitsky and Bartel 2013; Frankish et al. 2019). Despite being classified as noncoding, most lincRNAs contain open reading frames (ORFs) flanked by start and stop codons. Although many small ORFs encoding functional peptides have been identified recently within annotated human lincRNAs (Chen et al. 2020; Martinez et al. 2020; Ouspenskaia et al. 2021), the majority of lincRNAs peptide products have not been detected, and the mechanisms hindering their translation are unclear.

Sequencing of ribosome-protected fragments (Ribo-Seq) has highlighted differences between coding and noncoding RNAs ribosome interaction patterns, in particular concerning the tri-nucleotide periodicity of binding (Ji et al. 2015; Calviello et al. 2016) and ribosome release (Guttman et al. 2013). Furthermore, discriminating sequence features have also been noted between human mRNAs and lincRNAs (Niazi and Valadkhan 2012) and between lincRNAs with and without ribosome-association in human and mouse (Wang et al. 2017; Zeng and Hamada 2018). These studies identified a poor start codon context of lincRNA ORFs for translation initiation and reported cell-type-specific associations between human lincRNAs and ribosomes.

mRNA translation is regulated at initiation and during elongation (Tuller, Carmi, et al. 2010; Eraslan et al. 2019; Riba et al. 2019; Nieuwkoop et al. 2020). While RNA sequence and secondary structure around start codons are important for translation initiation, codon usage has been shown to affect translation elongation efficiency. Specifically, the rate of a codon’s translation correlates with the abundance of its cognate tRNA (Dana and Tuller 2014). Consequently, mRNAs composed of codons corresponding to more abundant tRNAs tend to be translated more efficiently. Evidence of such a mechanism to tune translation efficiency has been found in several contexts, for example, under cellular stress conditions, during proliferation and meiosis, and in cancer (Goodarzi et al. 2016; Torrent et al. 2018; Sabi and Tuller 2019; Guimaraes et al. 2020). The strength of the mRNA codon usage bias varies between species and is usually more pronounced in species under stronger selection pressure, that is, species with more efficient natural selection due to a larger effective population size or a shorter generation time (Subramanian 2008). Among eukaryotes, the mRNA codon usage bias is stronger in yeast and weaker in species such as human and mouse. In most species, the codon usage bias is also more pronounced for highly expressed mRNAs, likely because their sequences are under more intense selection pressure.

Given these well-established connections between mRNA sequence features and translation efficiency, the question arises whether lincRNAs simply just lack sequence features supporting efficient translation or whether they have evolved sequence features specifically hindering their translation. Moreover, if lincRNA sequence signatures were shaped through evolution, are those signatures stronger and more detectable in species whose genomes are under more intense selection, as is the case for sequence biases in mRNAs? To address these questions, we comprehensively analyzed sequence signatures indicative of hindered translation in lincRNAs and compared them with those in mRNAs and in neutrally evolving genomic sequences. We also compared the strengths of the lincRNA sequence signatures across five eukaryotes with various degrees of selection pressure and between all lincRNAs and those with the highest cytoplasmic expression levels. We further examined whether codon bias of lincRNAs could reduce their ribosome-binding in a cell-type-specific manner, using experimental data from five human cell lines.

## Results

### Open Reading Frames Are Frequent in lincRNAs

To investigate signatures in lincRNAs sequences decreasing translation efficiency, we focused on five species (*Homo sapiens*, *Mus musculus*, *Drosophila melanogaster*, *Caenorhabditis elegans*, and *Schizosaccharomyces pombe*). We selected these species because their genomes contain a sizable number (>1,000) of annotated lincRNA genes, and they are under a range of natural selection pressure, as seen in the strength of their mRNA codon usage bias (Subramanian 2008).

In order to exclude potential biases in the sequences of lincRNAs, we removed lincRNAs overlapping other genes, repetitive sequences (Jurka et al. 2005), and likely novel coding regions (predicted based on PhyloCSF score [Lin et al. 2011] or identified from Ribo-Seq data [Martinez et al. 2020]) (see “Materials and Methods”; supplementary table S1, Supplementary Material online). For each lincRNA, we extracted all ORFs longer than 30 nucleotides (=10 codons), starting with a canonical start codon (AUG) and ending at the first in-frame stop codon (UAG, UAA, UGA). To evaluate the lincRNA sequence bias in each species, we compared lincRNA signatures with those in likely neutrally evolving sequences. We chose nuclear (intronic) and non-transcribed (intergenic) sequences because these are not in contact with the translation machinery and therefore provide an unbiased reference. In particular, we analyzed ORFs in randomly selected intronic and intergenic regions of the same length and with the same G + C content (fraction of G and C nucleotides) as lincRNAs (see “Materials and Methods”). Since differences in the genomic sequence composition across species may affect the lincRNA measures, hindering their direct comparison, we only compared the deviations of lincRNAs from controls between species.

**Fig. 1.**
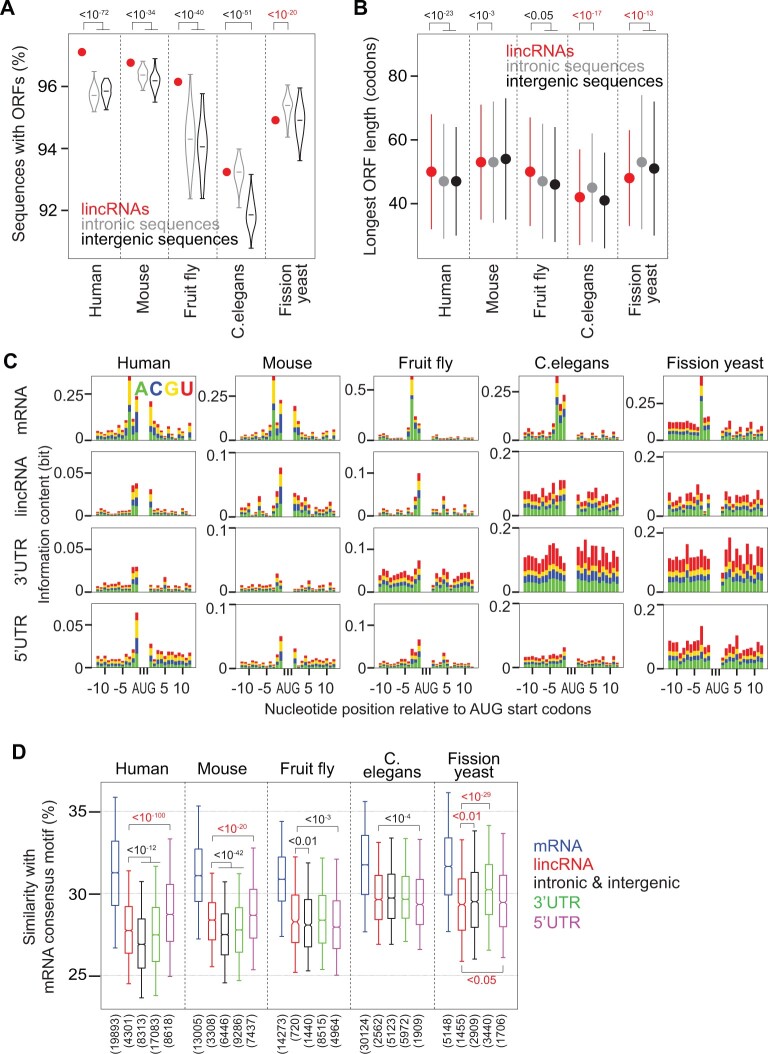
Prevalence, length, and start codon sequence context of lincRNA ORFs. (*A*) Percentage of lincRNA transcripts with ORFs (>10 codons; red dot) and percentage of random control regions with ORFs (in introns shown as gray and in intergenic regions shown as black violins), for five species. For each lincRNA transcript, 10 length- and G+C content-matched control sequences were randomly selected from introns and intergenic regions. *P* values are indicated from a one-sample *t*-test. (*B*) Median length of longest ORFs in lincRNAs (red), and intronic (gray) and intergenic (black) control sequences. Error bars represent median absolute deviations. *P* values are indicated from Wilcoxon’s rank-sum test. (*C*) Information content (see “Materials and Methods”) for the region ±12 nucleotides around AUG start codons for different ORFs (columns, indicated on top) and species (rows, indicated left). The sequence motif around mRNA start codons shows some similarity with the Kozak consensus sequence (gcc(A/G)ccAUGG). (*D*) Sequence similarity with the consensus mRNA sequence motif for the region ±12 nucleotides around start codons (see “Materials and Methods”) for mRNA coding regions (blue), lincRNA longest ORFs (red), longest ORFs in intronic and intergenic control sequences (black), and longest ORFs in 3′ UTRs (green) and 5′ UTRs (magenta). *P* values (<0.05) are indicated from Wilcoxon’s rank-sum test to compare lincRNAs with control regions, 3′ UTRs, or 5′ UTRs. *P* values are marked red if the median lincRNA value is below the value for the other region.

Most lincRNAs (>93% for each species) had at least one ORF (fig. 1*A*). Except for fission yeast, more lincRNAs than control regions contained ORFs. The number of ORFs per lincRNA was higher in all species, and its median ranged between 5 (fruit fly and *C.elegans*) and 9 (fission yeast) (supplementary fig. S1, Supplementary Material online). The longest ORFs (median length between 44 and 55 codons in different species) were significantly shorter in lincRNAs than in intronic and intergenic control regions for fission yeast and than in intronic regions for *C.elegans* (fig. 1*B*). Longest lincRNA ORFs were longer than longest control ORFs in mammals and fruit fly.

### RNA Sequence around lincRNA Start Codons Appears Suboptimal for Translation Initiation in Fission Yeast and C. elegans

For each gene, we only considered the mRNA isoform with the longest coding region, and the lincRNA isoform with the longest ORF (supplementary table S1, Supplementary Material online). From random intronic and intergenic regions of the same length as the lincRNA isoform harboring the longest ORF, we selected as control the longest ORF (>10 codons) with G + C content matching that of the longest lincRNA ORF. Additionally, we analyzed the longest ORF in coding genes’ 3′ untranslated regions (UTRs), which likely represent the least translated cytoplasmic RNA sequences (Guttman et al. 2013), and longest ORFs in 5′ UTRs of mRNAs, which were reported to resemble ribosome association with that of lincRNAs (Chew et al. 2013).

Since the sequence around start codons was identified as regulating translation in mRNA (Eraslan et al. 2019), we examined the sequence context around lincRNA start codons (fig. 1*C*). We found that in all species, the lincRNA sequence context was less similar to the consensus mRNA sequence motif, itself showing similarity to the Kozak sequence motif (Kozak 1989), than the individual sequence context around mRNA start codons (fig. 1*D*). Moreover, in fission yeast, this similarity was lower than the similarity of control ORFs and ORFs in 3′ UTRs and 5′ UTRs. There was no difference between lincRNAs and controls in *C.elegans*, whereas for mammals and fruit fly, the similarity for lincRNA was higher than for the controls. In mammals, 5′ UTRs were more similar to mRNAs than lincRNAs. Thus, the RNA sequence around lincRNA start codons appears less favorable for translation initiation in fission yeast but not in other species.

### Codon Composition of lincRNAs Is Distinct from mRNAs’ and Controls in Fission Yeast and C. elegans

**Fig. 2.**
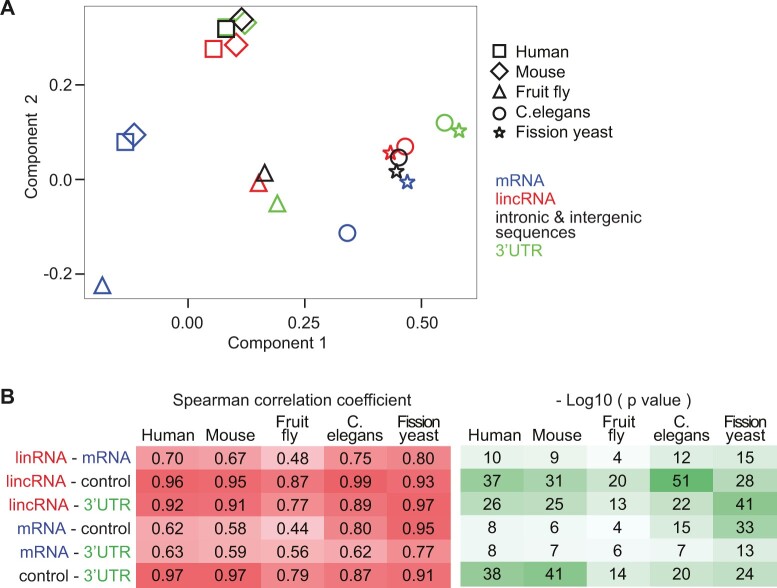
Comparison of trinucleotides (“codons”) in mRNA, lincRNA, control sequences, and 3′ UTRs. (*A*) First two components space from a multiple correspondence analysis performed on trinucleotide (“codon”) counts (excluding start and stop codons) in mRNA coding regions (blue) and longest ORFs in lincRNAs (red), intronic and intergenic control sequences (black), and 3′ UTRs (green) for five species. Results for 5′ UTRs are shown in supplementary figure S2, Supplementary Material online. (*B*) Spearman correlation coefficients between codon frequencies for the same ORFs and species as in (*A*).

mRNA codon usage is biased and contributes to translation regulation (Tuller, Waldman, et al. 2010; Hanson and Coller 2018). We investigated if biased frequencies of trinucleotides in lincRNAs—which for simplicity we refer to as codons—can contribute to decreasing translation efficiency. To gain global insight into codon usage in different RNA types, we performed a multiple correspondence analysis of their codon counts in different species (supplementary table S2, Supplementary Material online). We found that RNA types and species were clearly spread with orthogonal directions in the first two components (fig. 2*A*). In particular, fission yeast and *C.elegans* were grouped, as were both mammals. The fruit fly was equidistant to both groups.

Although for mammals and fruit fly, the codon composition of lincRNAs was positioned between mRNAs and controls, controls were closer to mRNA than lincRNA for *C.elegans* and fission yeast. In addition, 3′ UTR codon usage tended to be more distant from mRNA codon usage than lincRNAs’. These distinctions in codon usage between RNA types were reflected in the patterns of correlation strengths between codon frequencies of different RNA types (fig. 2*B*). In particular, for *C.elegans* and fission yeast, the correlation between mRNA and control codon frequencies was stronger than the correlation between lincRNA and mRNA codon frequencies.

### In Species under Strong Selection, Less Abundant tRNAs Are More Represented in lincRNA Codons Than in mRNA and Control Regions

**Fig. 3.**
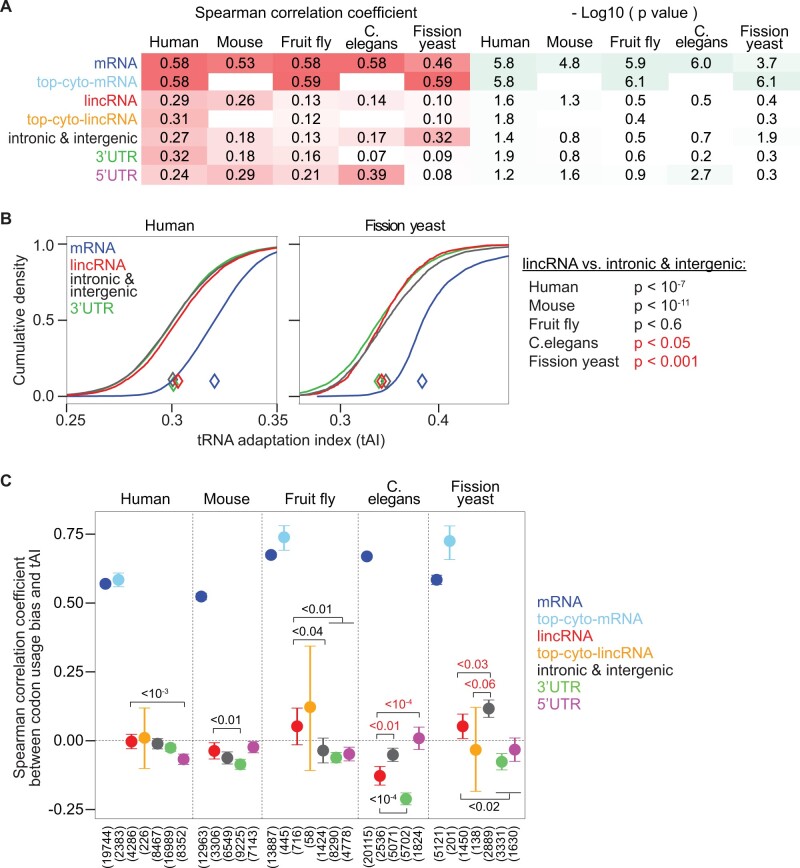
Correspondence between tRNA abundances and codon frequencies in different RNA types. (*A*) Spearman correlation coefficients between effective tRNA anticodon frequencies based on tRNA gene copy numbers (see “Materials and Methods”) and codon frequencies in mRNA coding regions and longest ORFs in lincRNAs, intronic, and intergenic control sequences, 3′ UTRs and 5′ UTRs for five species; and for codon frequencies in cytoplasmic mRNAs and lincRNAs for human, fruit fly, and fission yeast (see “Materials and Methods”). (*B*) Cumulative density of tAIs for mRNA coding regions (blue), and longest ORFs in lincRNAs (red), intronic and intergenic control sequences (black) and 3′ UTRs (green) for human (left panel) and fission yeast (right panel). Similar plots for other species and 5′ UTR ORFs are shown in supplementary figure S4, Supplementary Material online. On the right, *P* values from Wilcoxon’s rank-sum test to compare tAIs of lincRNAs with those of control sequences are indicated for all five species. *P* values are marked red if the median tAI of lincRNAs is lower than that of control sequences. (*C*) Translational selection test: Spearman correlation coefficient between tAI and codon usage bias (calculated as the KLD relative to the codon usage bias expected given the G+C content; see “Materials and Methods”) for ORF types and species as in (*A*). Error bars indicate 90% confidence intervals estimated from 10,000 times bootstrapping. *P* values are estimated as the fraction of times a correlation coefficient for lincRNAs is higher (indicated in red) or lower (indicated in black) than for controls or UTRs among 10,000 comparisons between bootstrapped samples. *P* values < 0.06 are indicated.

Next, we investigated how codon usages in different RNA types relate to tRNA abundances. As a first estimate of tRNA abundances in different species, we used the number of annotated tRNA genes for each tRNA anticodon type (see “Materials and Methods”), which correlates well with tRNA abundances (Tuller, Carmi, et al. 2010). We used wobble-base pairing and tRNA editing efficiencies (dos Reis et al. 2004) to estimate effective tRNA anticodon abundances for all codons, including those lacking a complementary tRNA encoded in the genome (see “Materials and Methods”). We found that mRNA codon frequencies correlated better with relative tRNA abundances than lincRNA codon frequencies for all species (fig. 3*A*). For fission yeast and fruit fly, the difference was more pronounced among highly expressed cytoplasmic mRNAs and lincRNAs (see “Materials and Methods”). For *C.elegans* and fission yeast—the two species with the strongest mRNA codon usage bias—lincRNA codon frequency correlated less with tRNA abundances than control codon frequency, suggesting a lincRNA codon usage bias towards codons corresponding to lower abundance tRNAs. On the contrary, in mammals, lincRNA codon frequencies are better correlated with tRNA abundances than controls. The correlations between tRNA abundances and codon frequencies for lincRNAs and controls were similar in fruit fly, locating this species in between the other groups again. Notably, the overall pattern of correlation strengths for different RNA types and species was similar among groups of codons with identical G + C content (supplementary fig. S3, Supplementary Material online), suggesting that differences in G + C content between RNA types and species are not causing the observed differences in correlations between codon and tRNA frequencies.

The tRNA adaptation index (tAI) is a measure for the correspondence between codon frequencies in an ORF and tRNA abundances (dos Reis et al. 2003). The tAI ranges from 0 to 1, where higher values indicate a preferential usage of codons decoded by more abundant tRNAs. For all species, mRNA coding regions had significantly higher tAI values (*P* < 10^−300^, Wilcoxon rank-sum test) than lincRNA ORFs (fig. 3*B*; supplementary fig. S4, Supplementary Material online). ORFs had significantly lower tAIs in lincRNAs than in control regions in *C.elegans* and fission yeast, whereas for mammals, tAIs were higher in lincRNAs than in controls (fig. 3*B*; supplementary fig. S4, Supplementary Material online). These tAI differences between lincRNA and controls align with the correlation analysis results (fig. 3*A*), potentially indicating that lincRNAs have adapted to use codons corresponding to less abundant tRNAs in fission yeast and *C.elegans*. Notably, the longest ORFs in 3′ UTRs also had lower tAIs than controls in fission yeast, *C.elegans*, and fruit fly, but similar tAIs as controls in mammals.

To further evaluate the extent and direction of codon usage bias in lincRNAs, we compared their tAIs with tAIs for trinucleotides in frameshifted ORFs. Such ORFs preserve the nucleotide content and sequence, including potential functional RNA sequence or structure motifs (see “Materials and Methods”). To account for underlying (di-)nucleotide biases in the genomic sequence of each species, we performed the same comparison with control region ORFs and used this as a reference. Overall, tAI differences (ΔtAI) between original and frameshifted ORFs were positive for mRNAs in all species (supplementary fig. S5, Supplementary Material online), indicating generally higher tAIs for the original coding sequences. ΔtAIs between original and frameshifted ORFs were closer to zero for other ORFs. In *C.elegans* and fission yeast, lincRNA ΔtAIs were significantly lower than controls, indicating that lincRNA tAIs tend to be lower than the frameshifted ones more often than for control ORFs. This strengthens the hypothesis that in *C.elegans* and fission yeast, lincRNA ORFs are biased for preferential usage of codons corresponding to less abundant tRNAs as opposed to maintaining specific RNA sequence or structure motifs. ΔtAIs for lincRNAs were overall larger than the controls in mouse.

dos Reis et al. (2004) proposed the correlation between tAI and the synonymous codon usage bias as a test for translational selection on mRNA coding regions in a species. Here, we modified this to test for a correlation between tAI and the overall codon usage bias (not just among synonymous codons; see “Materials and Methods”). This test confirmed that correlation coefficients were larger for mRNAs than for lincRNAs (*r* > 0.52 and *r* < 0.06, respectively, for all species), indicating a stronger adaptation of mRNA codon usage to tRNA abundances than for lincRNAs (fig. 3*C*). Furthermore, the correlation was higher among cytoplasmic mRNAs (*r* > 0.58) in human, fruit fly, and fission yeast, whereas it tended to be smaller among cytoplasmic lincRNAs (*r*<–0.03) in fission yeast. Strikingly, for *C.elegans* and fission yeast, lincRNA correlation coefficients were significantly lower than those for control ORFs (*P* < 0.03), and correlations were also significantly lower for 3′ UTR ORFs compared with control ORFs (*P* < 10^−4^, probability for a higher correlation coefficient for 3′ UTRs than for controls observed in 10,000 bootstrapped samples; see “Materials and Methods”). These results indicate that codons in noncoding ORFs are even less correlated with tRNA abundances than control codons in these species, potentially hindering translation. LincRNAs were also weaker correlated than 5′ UTRs in *C.elegans*, but stronger in human, fruit fly, and fission yeast.

### Cytoplasmic lincRNA Codons Correspond to Lower Expressed tRNAs Than Control Codons in Three out of Five Human Cell Lines, Concordant with Reduced Ribosome-Binding

In multicellular eukaryotes, tRNA expression is often tissue- and cell-type-specific (Dittmar et al. 2006; Pinkard et al. 2020), allowing to evaluate the impact of varying tRNA abundances on ribosome-binding to cytoplasmic lincRNAs. We focused on five human cell lines (GM12878, HEK293, HeLa-S3, HepG2, and K562), for which extensive experimental data are available to quantify relative tRNA expression levels, total and cytoplasmic lincRNA expression levels, and ribosome-binding to lincRNAs ([ENCODE Project Consortium 2004; Kishore et al. 2013; Subtelny et al. 2014; Cenik et al. 2015; Calviello et al. 2016; Aktaş et al. 2017; Solomon et al. 2017; Huang et al. 2019; Martinez et al. 2020]; see “Materials and Methods” and supplementary table S3, Supplementary Material online).

**Fig. 4.**
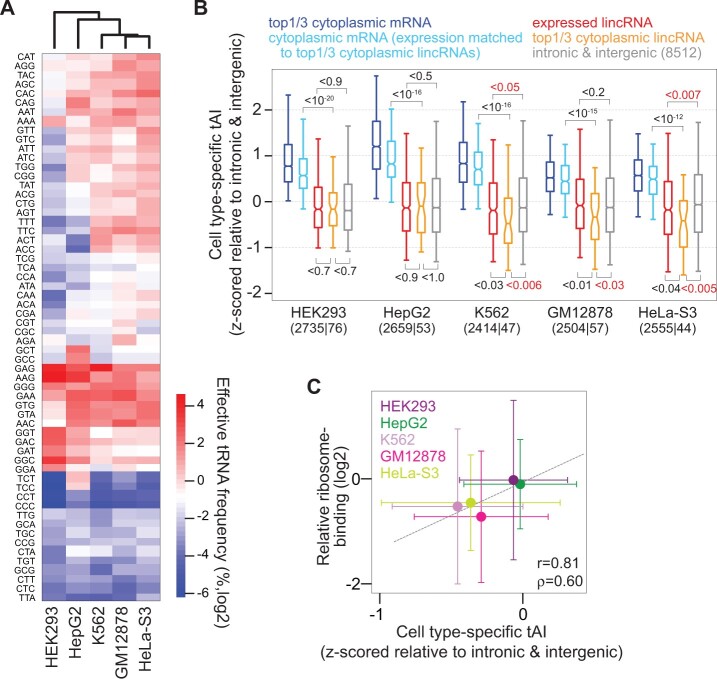
Cell-type-specific tAIs and ribosome-binding in five human cell lines. (*A*) Clustering (based on Euclidean distance) of effective tRNA anticodon frequencies, estimated from smallRNA-Seq data, in five human cell lines (indicated at bottom). (*B*) Boxplots of cell-type-specific tAIs (*z*-scored relative to those of intronic and intergenic control ORFs) for top-1/3 cytoplasmic expressed mRNAs (dark blue), mRNAs with cytoplasmic expression levels matching those of top-1/3 cytoplasmic lincRNAs (light blue), expressed lincRNAs (red), top-1/3 cytoplasmic expressed lincRNAs (orange), and intronic and intergenic control sequences (gray), for five human cell lines (label at bottom). The numbers of top-1/3 cytoplasmic mRNAs and lincRNAs are indicated in parentheses at the bottom. *P* values are calculated using Wilcoxson’s rank sum test. (*C*) Correspondence between cell-type-specific tAIs (*z*-scored relative to control tAIs) and relative ribosome-binding (see “Materials and Methods”) for 41 lincRNAs that are classified as cytoplasmic in all five human cell lines. Dots represent median values and error bars median absolute deviations. Pearson (*r*) and Spearman (*ρ*) correlation coefficients between median values are indicated.

tRNA abundances varied between cell lines (fig. 4*A*), with GM12878, HeLa-S3, and K562 showing relatively similar tRNA abundances (Spearman correlation >0.87), whereas those in HEK293 and HepG2 differed (Spearman correlation <0.75). Using these tRNA abundances, we calculated cell-line-specific tAIs (fig. 4*B*) and confirmed that abundant cytoplasmic lincRNAs had significantly lower tAIs than cytoplasmic mRNAs in all cell lines. Cytoplasmic lincRNA tAIs were also lower than tAIs of mRNAs with matching cytoplasmic expression levels (*P* < 10^−12^; see “Materials and Methods”). In the three cell lines showing similar tRNA abundances (GM12878, HeLa-S3, and K562), tAIs of abundant cytoplasmic lincRNAs were significantly lower than tAIs of control ORFs (*P* < 0.03) and than tAIs of all expressed lincRNAs (*P* < 0.04). For HEK293 and HepG2, tAIs of highly expressed cytoplasmic lincRNAs were not different from those of all expressed lincRNAs or control ORFs.

We next focused on lincRNAs classified as cytoplasmic in all five human cell lines (see “Materials and Methods”). tAIs for these 41 lincRNAs varied between cell lines and tended to be higher in HepG2 and HEK293 than in other cell lines. These differences in cell-line-specific tAIs were largely reflected in ribosome-binding differences (estimated from Ribo-Seq data; see “Materials and Methods”) between cell lines for the same 41 cytoplasmic lincRNAs (fig. 4*C*). In particular, ribosome-binding in HEK293 was not different from HepG2 and tended to be higher than for GM12878, HeLa-S3, and K562. Interestingly, increased lincRNA translation was reported before in the liver and kidney (van Heesch et al. 2019), although comparing between immortalized cell lines and primary cells from human tissues may be difficult. Thus, tAIs of abundant cytoplasmic lincRNAs were smaller than those of control ORFs in three out of five cell lines, concordant with reduced ribosome-binding in these cell lines.

### Ribosome-Binding Reflects tAI Differences between Cytoplasmic RNA Types, Particularly for Codons at the Beginning of ORFs

**Fig. 5.**
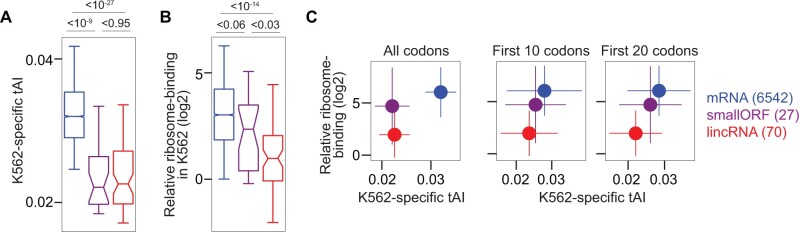
tAIs and ribosome-binding for coding RNAs and lincRNAs in K562 cells. (*A*) Cell-type-specific tAIs of mRNAs (blue), annotated lincRNAs with smORFs (purple) and lincRNAs (red). *P* values are indicated from Wilcoxon’s rank-sum test. RNAs are included that are classified as cytoplasmic and have Ribo-Seq reads mapped to the longest ORF. The number of RNAs of each type is identical for all subfigures and indicated in parentheses at the right of the figure. (*B*) Relative ribosome-binding (see “Materials and Methods”) to longest ORFs. *P* values are indicated from Wilcoxon’s rank-sum test. (*C*) Correspondence between K562-specific tAIs (*x*-axis) and relative ribosome-binding (*y*-axis) for three types of cytoplasmic RNAs. tAIs are calculated for all codons (first panel), the first 10 codons (second panel) and the first 20 codons after ORF starts (last panel). Dots show the median values and error bars the median absolute deviation.

To further understand the relationship between tAI and ribosome-binding, we focused on three types of cytoplasmic RNAs: mRNAs, lincRNAs, and annotated lincRNAs with experimentally validated small protein-encoding ORFs (smORFs; see “Materials and Methods”). We observed that differences in tAIs between RNA types were mostly concordant with differences in ribosome-binding, estimated from Ribo-Seq data. In particular, tAIs of lincRNAs and smORFs were significantly different from those of mRNAs (fig. 5*A*), and relative ribosome-binding, which accounts for differences in ORF length and expression between RNA types (see “Materials and Methods”), was significantly lower for lincRNAs than for other cytoplasmic RNA types, for instance in K562 (fig. 5*B*).

However, differences in relative ribosome-binding between lincRNAs and smORFs were more pronounced than their differences in tAIs (fig. 5*C*, first panel). Previously, the codon usage immediately downstream of mRNA start codons was proposed to play a specific role in facilitating translation initiation and thereby contributing to efficient translation elongation (Tuller, Carmi, et al. 2010; Bentele et al. 2013). Thus, we investigated if tAIs calculated for the first codons downstream of start codons in different RNA types may better agree with the observed ribosome-binding differences. Indeed, tAIs calculated for the first 10 or 20 codons tended to match better the differences in ribosome-binding between RNA types in K562 cells (fig. 5*C*, last two panels) and in almost all other cell lines (supplementary fig. S6, Supplementary Material online, last two rows). We found no indication for the RNA sequence or structure context around start codons of smORFs and lincRNA ORFs to explain the observed ribosome-binding differences between these cytoplasmic RNA types (data not shown).

These observations suggest that the first ORF codons in cytoplasmic lincRNAs can influence ribosome-binding, potentially through impeding translation initiation.

## Discussion

In the absence of any functional role of the peptides resulting from lincRNA translation, it would likely be advantageous to reduce stable associations between lincRNAs and ribosomes for several reasons: unwanted lincRNA translation wastes energy (Wagner 2005), reduces the pool of ribosomes available for mRNA translation (Raveh et al. 2016), and may lead to the synthesis of peptides with possibly harmful interference. Furthermore, it may hinder a potential regulatory function of lincRNAs, and, given the association between mRNA translation and transcript stability (Presnyak et al. 2015; Tuck et al. 2020), translation of lincRNAs might impact their cytoplasmic expression levels, with potentially disadvantageous consequences.

**Fig. 6.**
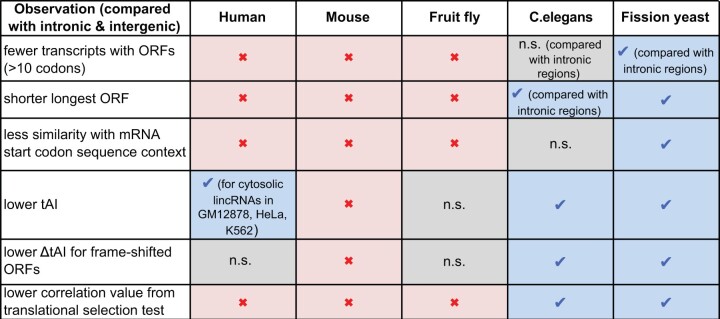
Summary of the observed signatures of hindered lincRNA translation in five species. Blue check marks indicate that the corresponding measure is significantly lower for lincRNAs compared with intronic and intergenic control sequences, n.s. stands for not significant when there was a trend for a lower measure in lincRNAs, and a red cross indicates that the corresponding measure is significantly higher for lincRNAs compared with controls.

We analyzed signatures of repressed or inefficient translation in lincRNA sequences from five eukaryotes (summarized in fig. 6). The analyzed signatures included general properties such as the prevalence and length of ORFs, signatures related to translation initiation such as the sequence context around start codons, and signatures indicative of efficient elongation such as the codon usage and its relation with tRNA abundances. To evaluate the specificity of the latter signatures, we compared them with those in frameshifted ORFs and performed a translational selection test. Although all analyzed signatures in lincRNAs were markedly different from those in mRNAs, for all species, all lincRNA signatures in fission yeast and most signatures (except fewer ORFs and less efficient translation initiation) in *C.elegans* were also stronger than in intronic and intergenic control sequences.

These differences in lincRNA sequence signatures across species are consistent with a stronger selection pressure (or more efficient selection) on the sequences of lincRNAs in fission yeast and *C.elegans* than in other eukaryotes, where translational selection on mRNA was also found to be weaker (dos Reis and Wernisch 2009).

We acknowledge that, although statistically significant, some of the observed effect sizes of the signatures hindering translation in fission yeast and *C.elegans* are relatively small. However, the consistency of these effects across multiple, largely independent, signatures substantiates the hypothesis that lincRNA sequences are biased to hinder translation in these species. Moreover, because of their lower expression level—likely connected to lower selection pressure—sequence biases in lincRNAs are expected to be smaller than in mRNAs.

The lack of observed signatures counteracting translation (in particular those related to translation initiation and ORF occurrence) in species such as human and mouse may also suggest that lincRNA translation is not universally repressed in these species but to be so in a more cell-type-specific manner. Indeed, estimating cell-type-specific tRNA expression levels, we observed for three out of five human cell lines that codons in abundant cytoplasmic lincRNAs are less correlated to the expressed tRNAs than trinucleotides in control ORFs (fig. 4*B*). Such a result is consistent with previous reports of cell-type- or condition-specific translation of human lincRNAs (Wang et al. 2017; van Heesch et al. 2019; Chen et al. 2020; Martinez et al. 2020; Ouspenskaia et al. 2021), potentially because some peptides cause no harm or even serve a function in specific cell types or under certain conditions.

Interestingly, we detected similar signatures hindering translation in the longest ORFs of 3′ UTRs, and the translational selection test revealed lowest correlation values for 3′ UTRs for all species (fig. 3*C*). Several signatures were even slightly stronger for 3′ UTRs than for lincRNAs, such as the difference of codon usage with mRNAs for *C.elegans* and fission yeast (fig. 2*A*), or lower tAIs for *C.elegans*, fission yeast, and fruit fly (supplementary fig. S4, Supplementary Material online). These signatures could result from a stronger selection pressure on 3′ UTRs than on lincRNA ORFs, potentially because most 3′ UTRs are more expressed in the cytoplasm and are evolutionarily older (Ulitsky and Bartel 2013) and thus had more time for adapting their sequence to tRNA abundances. The fact that 3′ UTR tAIs for mammals were not lower than control tAIs (supplementary fig. S4, Supplementary Material online) might indicate that, in these species, the selection pressure on noncoding ORFs is not sufficiently strong to result in tAIs lower than for control ORFs, or that cell-type-specific translation regulation is more relevant. Longest ORFs in 5′ UTRs, in contrast, showed a better start codon context for translation initiation in mammals, likely indicating a frequent functional role of upstream ORFs in these species.

We explored the functional impact of varying tRNA expression levels on ribosome-binding to lincRNAs by analyzing cell-type-specific tAIs and ribosome-binding to cytoplasmic lincRNAs in five human cell lines. We propose a mechanistic link between tAI and ribosome-binding to lincRNA ORFs that would allow cell-type-specific hindering of lincRNA translation. tAIs for the first 10–20 codons of cytoplasmic ORFs appeared to better match differences in ribosome-binding between coding and noncoding RNA types in several cell lines, suggesting that codons at the start of ORFs are particularly important for hindering ribosome engagement and translation initiation (or promoting it in case translation would be advantageous).

This study provides a comprehensive analysis of signatures hindering efficient translation in lincRNA sequences of five species and five human cell lines. Although the analyzed species are widely studied model organisms, several recently identified lincRNAs (Akay et al. 2019) and peptide-coding lincRNAs (Martinez et al. 2020; Ouspenskaia et al. 2021) indicate that the annotation of lincRNAs and their translation status may not yet be complete. We believe that with more accurate lincRNA annotations our identified sequence signatures will become stronger, and they may help to distinguish genuine lincRNAs with regulatory roles in the cytoplasm from those coding for peptides in a cell-type- or condition-specific manner. An interesting aspect is how cell-type- and condition-specific tRNA expression imposes different constraints on the evolution of cytoplasmic lincRNA sequences to either curb ribosome-binding in specific cell types or promote it to enable peptide translation in others. Analyzing the impact of natural genetic variation or targeted mutations in lincRNA sequences on ribosome-binding and peptide translation might shed light on these questions.

## Materials and Methods

### Gene Annotations

We downloaded gene annotations and genomic sequences from GENCODE (Frankish et al. 2019) (www.gencodegenes.org, last accessed December 8, 2021) for *Homo sapiens* (v19 corresponding to hg19) and *Mus musculus* (vM16 corresponding to mm10), from Ensembl (www.ensembl.org, last accessed December 8, 2021) for *D.melanogaster* (dm6) and *C. elegans* (ce11), and from EnsemblFungi (http://fungi.ensembl.org, last accessed December 8, 2021) for *S.pombe* (ASM294v2). For *C.elegans*, we also included lincRNA genes recently identified by Akay et al. (2019) and not contained the Ensembl gene annotations. We chose fission yeast as opposed to the more commonly studied budding yeast because the number of annotated lincRNA genes is much larger in fission yeast (>1,000) than in budding yeast (<100). For all species, we excluded genes on mitochondrial chromosomes from our analysis as these are translated using mitochondrial tRNAs.

### mRNA Coding Regions

For each mRNA gene we analyzed only the longest coding region starting with a canonical start codon (AUG), from all coding regions present in different transcript isoforms.

### Identification of Open Reading Frames in lincRNAs

We excluded lincRNA genes overlapping other gene annotations by at least one nucleotide on either strand, those overlapping regions of high PhyloCSF score (Lin et al. 2011) (PhyloCSF Novel tracks downloaded from https://data.broadinstitute.org/compbio1/PhyloCSFtracks/, last accessed December 8, 2021 for human, mouse, fruit fly, and *C.elegans*), and, in case of human, those overlapping regions experimentally identified to encode small proteins based on Ribo-Seq data in human cell lines (supplementary table 1 of Martinez et al. 2020). Furthermore, we excluded lincRNA genes with exons that overlapped (by more than 30 nucleotides) with simple repeats or low complexity regions (repeat masker annotations downloaded for human, mouse, fruit fly, and *C.elegans* from http://hgdownload.soe.ucsc.edu/goldenPath/, last accessed December 8, 2021), as such regions might bias the sequence composition of lincRNAs. We list the number of remaining lincRNAs after all these filtering steps in supplementary table S1, Supplementary Material online. We identified ORFs longer than 30 nucleotides (=10 codons) that start with a canonical start codon (AUG) and end at the first in-frame stop codon (UAG, UAA, UGA) and for each lincRNA gene kept only the transcript isoform harboring the longest ORF for further analysis.

### Intronic and Intergenic Control Sequences

As controls, we considered nuclear (intronic) and non-transcribed (intergenic) sequences. We took intronic regions from mRNA genes and excluded the ten nucleotides flanking exons on each side. The chosen intergenic regions did not overlap any gene annotation on either strand. We excluded intronic and intergenic regions that overlapped with likely novel coding regions (based on PhyloCSF novel track for human, mouse, fruit fly, and *C.elegans*, and Ribo-Seq data for human) and with repetitive sequence regions (from repeat masker annotations for human, mouse, fruit fly, and *C.elegans*). For each selected lincRNA transcript, a control sequence of the same length was randomly selected from intronic and intergenic regions using bedtools shuffle (Quinlan and Hall 2010). In each control sequence, we identified the longest ORF (>10 codons). If the fraction of G and C nucleotides (rounded to two decimal places) matched the lincRNA ORF’s, we retained the sequence as a control for that lincRNA; otherwise, we repeated the random selection until a sequence with equal G + C content was found.

To compare ORF identification in lincRNAs and control sequences (fig. 1*A* and *B*), we randomly selected, for each lincRNA transcript, ten length- and G + C content-matched control sequences from intronic and from intergenic regions.

### *Longest ORFs in 3*′ *UTRs and 5*′ *UTRs*

We defined ORFs (>10 codons) in 3′ UTRs and 5′ UTRs of coding genes from a canonical AUG start codon to the first in-frame stop codon. For each gene, we considered in our analysis only the longest ORF out of ORFs in 3′ UTRs (or 5′ UTRs) of different isoforms.

### Small Protein-Encoding ORFs

The human smORFs we used were the longest ORFs in annotated lincRNAs that overlapped (by at least one nucleotide on the same strand) with a small protein-encoding region, identified experimentally by Martinez et al. in three human cell lines based on Ribo-Seq data (supplementary table 1 of Martinez et al. 2020), or with a region with high PhyloCSF score (Lin et al. 2011) (downloaded from https://data.broadinstitute.org/compbio1/PhyloCSFtracks/, last accessed December 8, 2021 for human).

### Sequence Context around Start Codons

To analyze the sequence context around start codons, we counted the fraction of nucleotides (A, C, G, T) at each position in the region ±12 nucleotides around. We calculated the information content (*I*) at each position as: $I=2+\sum_{n=A,C,G,T}^{}f_{n}\;\log2(f_{n})$, where $f_{n}$ is the frequency of nucleotide *n*. The probability (*P*) for the consensus mRNA start codon motif (also referred to as similarity) was calculated as: $P=\exp\{\left[\sum_{l=-12}^{12}\log(f_{n\left(l\right)})]/L\right)\}$, where *L* is the total length of the start codon region, and $f_{n(l)}$ is the frequency of nucleotide *n* (for mRNA) at position *l* around the start codon of an ORF.

### Estimation of Relative tRNA Abundances Based on tRNA Gene Counts

We downloaded tRNA gene predictions from GtRNAdb (http://gtrnadb.ucsc.edu/GtRNAdb2/, last accessed December 8, 2021) (Chan and Lowe 2016) for all species studied. We counted the number of annotated tRNA genes coding for the same tRNA anticodon type taking into account high confidence tRNA gene predictions. We calculated effective tRNA abundances using previously determined weights to account for the contributions of tRNA editing and wobble-base pairing at the first tRNA anticodon position (dos Reis et al. 2004). Specifically, the weights *w* were *w*(G:U)=0.41, *w*(I:C)=0.28, *w*(I: A)=0.9999, and *w*(U:G)=0.68, where the first letter denotes the first nucleotide of a tRNA anticodon nucleotide triplet and the second letter the third nucleotide of a codon. We then calculated effective tRNA abundances as:

$${\left(tRNA_{NNU}\right)}_{eff}=tRNA_{NNU}+[1-\text{w}(\text{G}:\text{U})]*tRNA_{NNC}{\left(tRNA_{NNC}\right)}_{eff}=tRNA_{NNC}+[1-\text{w}(\text{I}:\text{C})]*tRNA_{NNU}{\left(tRNA_{NNA}\right)}_{eff}=tRNA_{NNA}+[1-\text{w}(\text{I}:\text{A})]*tRNA_{NNU}{\left(tRNA_{NNG}\right)}_{eff}=tRNA_{NNG}+[1-\text{w}(\text{U}:\text{G})]*tRNA_{NNA}$$

The above nucleotide triplets are the corresponding codon sequences (i.e. the reverse complements of the tRNA anticodon sequences). N stands for any nucleotide.

### Estimation of Cell-Type-Specific tRNA Abundances

Due to the repetitive nature of tRNAs, their strong secondary structure, and the high frequency of posttranscriptional tRNA modifications, high-throughput experimental quantification of tRNA expression levels is challenging. Two dedicated experimental high-throughput approaches for the quantification of tRNA expression, hydro-tRNA-Seq (Gogakos et al. 2017) and DM-tRNA-Seq (Zheng et al. 2015), have been proposed and were applied in human HEK293 cells. smallRNA-Seq was also used previously to quantify tRNA expression (Gingold et al. 2014; Ji et al. 2015; Hernandez-Alias et al. 2020), and these data are more widely available for different human cell types. Thus, we used smallRNA-Seq-based tRNA abundances to calculate cell-type-specific tAIs for all cell lines, as follows.

Fastq files with smallRNA-Seq reads were downloaded for different cell types from various sources (see supplementary table S3, Supplementary Material online). Reads were preprocessed using the fastx toolkit (http://hannonlab.cshl.edu/fastx_toolkit/, last accessed December 8, 2021) and then mapped to native and mature tRNA sequences using segemehl v0.2 (Hoffmann et al. 2009). Of the mapped reads, only those with a minimum length of 15 nucleotides were retained. To account for the high frequency of tRNA modifications, which may result in mapping mismatches, the allowed mismatch ratio (mismatched nucleotides/read length) was set to ≤10%. (Other mismatch ratio cutoffs, <7% and <15%, were also tested for HEK293, but did not improve the correlation with tRNA abundances derived from hydro-tRNA-Seq [Gogakos et al. 2017] and DM-tRNA-Seq [Zheng et al. 2015] data, or resulted in a smaller fraction of reads mapping to tRNA sequences in sense direction; data not shown.) tRNA abundances were calculated as the number of smallRNA-Seq reads mapping to each tRNA anticodon type. Effective tRNA abundances are calculated from these as described above.

### tRNA Adaptation Index

As proposed by dos Reis et al. (2004), we calculated ORF tAIs as the geometric mean of normalized effective tRNA abundances complementary to codons in the ORF:

$$\mathrm{tAI}=\sqrt[{n}]{\prod_{i}^{n}w_{i}},$$

where n is the total number of codons in an ORF and $w_{i}$ is the normalized effective tRNA abundance of the tRNA anticodon complementary to the codon at position i.

We obtained normalized tRNA abundances by dividing each effective tRNA abundance by the maximum of all effective tRNA abundances:

$$w_{i}=\frac{f_{\mathrm{tRN}A_{i}}}{\max\left(f_{\mathrm{tRN}A_{i}}\right)},$$

where $f_{\mathrm{tRN}A_{i}}$ is the frequency of the tRNA complementary to the codon at position i.

We chose to normalize to the maximum of all effective tRNA abundances instead of the maximum among synonymous codons coding for the same amino acid because we wanted to analyze the global correspondence between tRNA abundances and codon usage, independent of amino acid identities.

We calculated first-10-codon and first-20-codon tAIs by considering only the first 10 and 20 codons downstream of the AUG start, respectively.

### Randomized Control Sequences

Frameshifted tAIs were calculated from codon frequencies in sequences starting one and two nucleotides downstream of start codons and ending two and one, respectively, nucleotides upstream of stop codons of ORFs.

### Modification of the Correlation Test for Translational Selection from dos Reis et al. (2004)

To test for translational selection on mRNA codon usage in a species, dos Reis et al. (2004) proposed a correlation test, in particular the correlation between tAI and the effective number of codons, adjusted for the G + C bias at the third codon position of mRNAs. Here, we modified this translational selection test to quantify the global correspondence between codon usage bias and tRNA abundances for all 60 codons (excluding start and stop codons), not just the correspondence within groups of synonymous codons. For that, we used the Kullback–Leibler divergence (KLD), which was used before to quantify codon usage bias (Bentele et al. 2013). KLD is the relative entropy between the actual codon usage in an ORF and the codon usage that would be expected based on its G + C content:

$$\mathrm{KLD}=-\sum_{c}^{}{\;p}_{\mathrm{obs}}\left(c\right){\;\log}_{2}\left(\frac{p_{\mathrm{obs}}\left(c\right)}{p_{\exp}\left(c\right)}\right).$$

${\;p}_{\mathrm{obs}}(c)$ is the observed frequency of codon c among all codons used in an ORF, and $p_{\exp}(c)$ is the expected frequency of codon c, given by the G + C content, $f_{\mathrm{GC}}$, of an ORF and normed to 1 for all codons cc occurring in an ORF:

$$p_{\exp}(c)={(\left(f_{\mathrm{GC}}\right)}^{n_{\mathrm{GC}}\left(c\right)}{\left(1-f_{\mathrm{GC}}\right)}^{n_{\mathrm{AT}}\left(c\right)})/(\sum_{cc}^{}{\left(f_{\mathrm{GC}}\right)}^{n_{\mathrm{GC}}\left(cc\right)}{\left(1-f_{\mathrm{GC}}\right)}^{n_{\mathrm{AT}}\left(cc\right)}).$$

$n_{\mathrm{GC}}(c)$ and $n_{\mathrm{AT}}(c)$ are the numbers of G or C and A or T nucleotides, respectively, in codon c. We used the Spearman correlation coefficient between (the negative) KLD and tAI as a measure for the strength of translational selection. We estimated the correlation coefficient confidence intervals (90%) in figure 3*C* by 10,000 times bootstrapping with replacement. *P* values indicate the probability for a higher or equal correlation coefficient for lincRNAs (indicated in red) or a lower correlation for lincRNAs (indicated in black) compared with control ORFs, 3′ UTR, or 5′ UTR ORFs among 10,000 bootstrapped samples.

### Quantification of Cytoplasmic Gene Expression Levels per Species

We estimated cytoplasmic RNA expression levels for three species (human, fruit fly, and fission yeast) by combining cytosolic gene expression quantifications from different studies (see supplementary table S3, Supplementary Material online, for sources and accession codes). For fruit fly, we used data from early embryos and two cell lines, S2 and ML-DmD17-c3 (Aspden et al. 2014; Li et al. 2016; Bouvrette et al. 2018). For fission yeast, we used data from two different growth conditions (Subtelny et al. 2014; Herzel et al. 2018). For humans, we used data from five cell lines, GM12878, HEK293, HeLa-S3, HepG2, and K562 (ENCODE Project Consortium 2004; Subtelny et al. 2014). For GM12878, HeLa-S3, HepG2, and K562, we downloaded gene quantifications from ENCODE (www.encodeproject.org, last accessed December 8, 2021). For HEK293 and the other species, we downloaded fastq files with polyA-selected RNA-Seq reads obtained from cytosolic RNA fractions, preprocessed them using the fastx toolkit (http://hannonlab.cshl.edu/fastx_toolkit/, last accessed December 8, 2021), and quantified gene expression levels using RSEM (Li and Dewey 2011) with STAR (Dobin et al. 2013). We averaged gene expression levels over replicates. To combine expression data from different experiments for each species, we first ranked genes by their cytoplasmic expression level in each experiment. We then used the maximum rank across experiments as the combined rank for each gene. Combined ranks were normalized to the maximum combined rank so that genes with the highest cytosolic expression levels had combined ranks close to 1. We defined lincRNAs with a combined rank > 0.85 as top cytoplasmic lincRNAs, and mRNAs with a combined rank > 0.99 as top cytoplasmic mRNAs.

### Quantification of Total and Cytosolic Transcript Expression Levels in Human Cell Lines

We used total and cytoplasmic transcript expression levels for five human cell lines (GM12878, HEK293, HeLa-S3, HepG2, and K562; see supplementary table S3, Supplementary Material online, for sources and accession codes). We downloaded transcript quantifications for four cell lines from ENCODE (ENCODE Project Consortium 2004). For HEK293, we downloaded polyA-selected RNA-Seq reads (Subtelny et al. 2014; Aktaş et al. 2017) and preprocessed them using the fastx toolkit (http://hannonlab.cshl.edu/fastx_toolkit/, last accessed December 8, 2021). We quantified transcript expression levels using RSEM (Li and Dewey 2011) with STAR (Dobin et al. 2013). We averaged expression levels over replicates. We considered RNAs with TPM > 0.1 as expressed. We chose the threshold for defining cytoplasmic RNAs as the first-quartile (25%) of the mRNA cytosolic expression values for each cell type. We excluded histone mRNAs, as these are not usually polyadenylated and result in incorrect expression values in polyA-selected RNA-Seq.

We selected mRNAs with cytoplasmic expression levels matching those of lincRNAs as follows. First, for each cytoplasmic lincRNA, we identified all mRNAs with similar cytoplasmic expression levels (identical values after log10-transformation and rounding to two decimal places). From these mRNAs, we randomly sampled ten with replacement for each cytoplasmic lincRNA.

### Quantification and Analysis of Ribo-Seq Data in Human Cell Lines

We downloaded Ribo-Seq data for five human cell lines from several studies ([Subtelny et al. 2014; Cenik et al. 2015; Solomon et al. 2017; Huang et al. 2019; Martinez et al. 2020]; see supplementary table S3, Supplementary Material online). We trimmed adapter sequences from reads' ends using cutadapt v1.8 (Martin 2011), and retained reads with a length between 16 and 35 nucleotides and a quality score of ≥30 in at least 90% of the bases. We discarded reads that mapped to human rRNAs or tRNAs (ENSEMBL database v91 [Zerbino et al. 2018]) using bowtie2 v2.3.0 (-L 15 -k 20) (Langmead and Salzberg 2012). We further discarded reads that mapped to two or more mRNA coding regions or longest lincRNA ORFs.

We determined the position of the ribosome P site within Ribo-Seq reads from reads overlapping mRNA start codons. In particular, we considered the three most frequent distances of the AUG start codon from the read start (read offset) for each read length (if they were found in more than 500 reads and more than 1% of reads overlapping mRNA start codons). Then, for each longest mRNA coding region or longest lincRNA ORF, we counted Ribo-Seq reads if the corresponding ribosome P site was in-frame, considering the three read offsets for that respective Ribo-Seq read length.

We calculated the relative ribosome-binding for each longest coding region/ORF as the log2 ratio of the normalized Ribo-Seq read count (plus a pseudo-count of 1.0) to the cytosolic expression level (FPKM) of the transcript harboring the longest coding region/ORF. We normalized the Ribo-Seq read counts by the length of the coding region/ORF and the total number of Ribo-Seq reads mapping in-frame to mRNA coding regions, and multiplied by 1e + 9.

We excluded histone mRNAs from this analysis, as these are not usually polyadenylated and result in incorrect expression values in polyA-selected RNA-Seq and snoRNAs, as these may associate with ribosomes that translate other RNAs.

## Supplementary Material

Supplementary data are available at *Molecular Biology and Evolution* online.

## Supplementary Material

msab356_Supplementary_Data
