# Supplementary material for: Analysis of Eukaryotic lincRNA Sequences Indicates Signatures of Hindered Translation Linked to Selection Pressure
Source: Mol Biol Evol. 2021 Dec 13;39(2):msab356. doi: 10.1093/molbev/msab356 (PMC8826458; doi:10.1093/molbev/msab356)
Supplement: msab356_Supplementary_Data [file msab356_supplementary_data.zip › SupplementaryFiguresS1-S6.pdf]

Figure S1

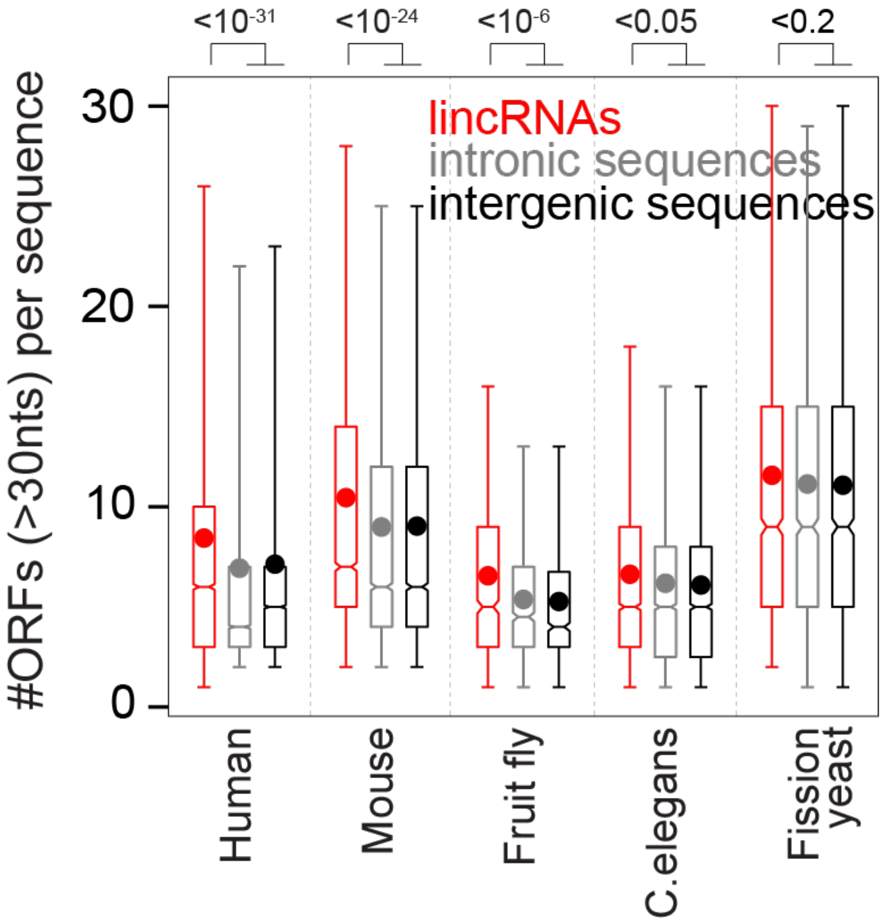

Figure S1: Number of ORFs (>10 codons) in lincRNAs and control sequences for five species

Boxplots of number of ORFs (>10 codons) in lincRNAs (red) and in intronic (gray) and intergenic (black) control sequences for different species. Dots indicate the average number of ORFs per sequence. P values are from Wilcoxon's Rank sum test.

Figure S2

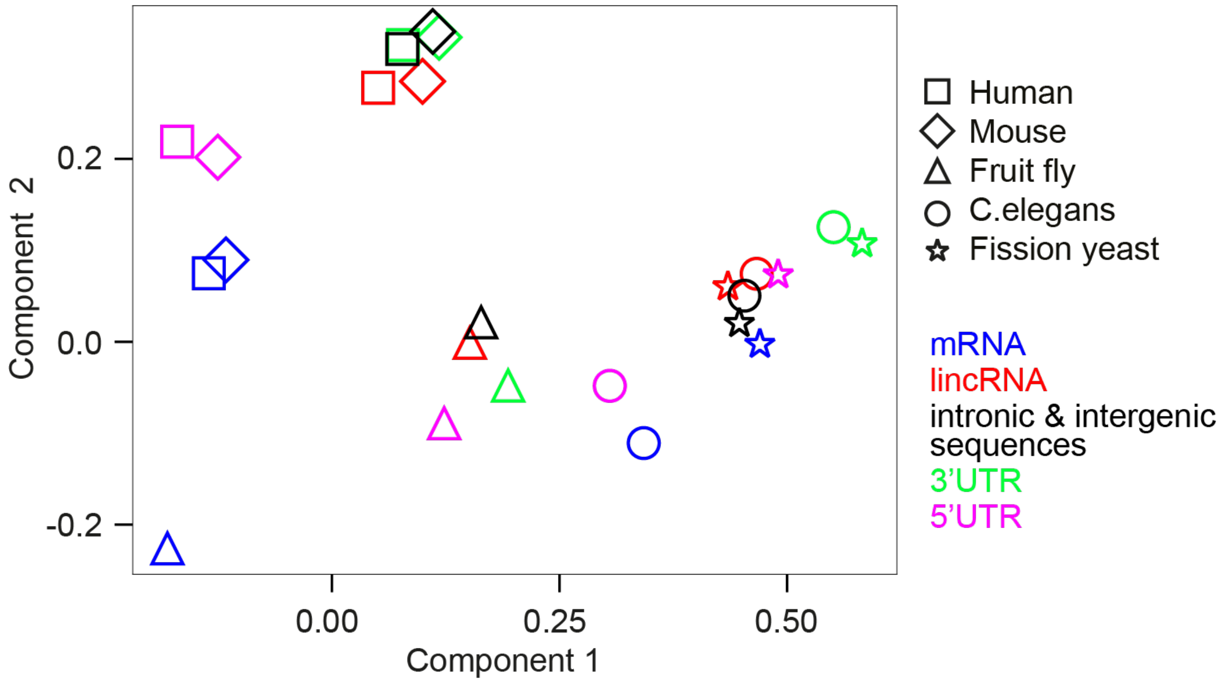

Figure S2: Comparison of trinucleotides (“codons”) in mRNA, lincRNA, control sequences, 3’UTRs and 5’UTRs.

First two components space from a multiple correspondence analysis performed on trinucleotide (“codon”) counts (excluding start and stop codons) in mRNA coding regions (blue) and longest ORFs in lincRNAs (red), intronic and intergenic control sequences (black), 3’UTRs (green), and 5’UTRs (magenta) for five species.

Figure S3

	Spearman correlation coefficient					- Log10 (p value)				
	Human	Mouse	Fruit fly	C elegans	Fission yeast	Human	Mouse	Fruit fly	C elegans	Fission yeast
	G+C=0 (7 codons)					G+C=0 (7 codons)				
mRNA	0.68	0.75	0.72	0.89	0.86	1.0	1.3	1.2	2.2	1.9
top-mRNA	0.68		0.76		0.86	1.0		1.3		1.9
lincRNA	0.57	0.57	0.36	0.46	0.57	0.7	0.7	0.4	0.5	0.7
top-lincRNA	0.46		0.50		0.52	0.5		0.6		0.6
control	0.39	0.57	0.50	0.46	0.68	0.4	0.7	0.6	0.5	1.0
3'UTR	0.46	0.50	0.58	0.61	0.50	0.5	0.6	0.8	0.8	0.6
5'UTR	0.46	0.50	0.36	0.71	0.50	0.5	0.6	0.4	1.1	0.6
	G+C=1 (21 codons)					G+C=1 (21 codons)				
mRNA	0.55	0.57	0.68	0.68	0.57	2.0	2.1	3.2	3.0	2.2
top-mRNA	0.54		0.69		0.62	2.0		3.2		2.6
lincRNA	0.04	0.11	0.18	0.18	0.34	0.1	0.2	0.4	0.4	0.9
top-lincRNA	0.02		0.14		0.27	0.0		0.3		0.6
control	-0.01	0.04	0.17	0.27	0.61	0.0	0.1	0.3	0.6	2.5
3'UTR	0.16	0.07	0.52	0.08	0.41	0.3	0.1	1.8	0.1	1.2
5'UTR	0.15	0.09	0.36	0.38	0.44	0.3	0.2	1.0	1.1	1.3
	G+C=2 (24 codons)					G+C=2 (24 codons)				
mRNA	0.52	0.47	0.27	0.48	0.61	2.0	1.7	0.7	1.8	2.8
top-mRNA	0.59		0.28		0.75	2.6		0.7		4.5
lincRNA	0.37	0.38	0.02	0.13	0.00	1.1	0.8	0.0	0.3	0.0
top-lincRNA	0.32		0.04		-0.12	0.9		0.1		0.2
control	0.38	0.31	0.00	0.27	0.57	1.2	0.8	0.0	0.7	2.4
3'UTR	0.39	0.29	0.07	0.02	-0.04	1.2	0.8	0.1	0.0	0.1
5'UTR	0.37	0.33	0.08	0.38	-0.05	1.1	0.9	0.1	1.2	0.1
	G+C=3 (8 codons)					G+C=3 (8 codons)				
mRNA	0.69	0.25	0.90	0.53	0.87	1.2	0.3	2.7	0.7	2.3
top-mRNA	0.69		0.95		0.84	1.2		3.6		2.0
lincRNA	0.62	0.01	0.43	0.41	0.81	1.0	0.0	0.5	0.5	1.9
top-lincRNA	0.62		-0.14		0.60	1.0		0.1		0.9
control	0.52	-0.10	0.55	0.37	0.91	0.7	0.1	0.8	0.4	2.8
3'UTR	0.48	-0.10	0.90	-0.16	0.71	0.6	0.1	2.7	0.1	1.3
5'UTR	0.71	0.30	0.74	-0.08	0.37	1.3	0.3	1.4	0.1	0.4

Figure S3: Correlation between codon and tRNA frequencies for groups of codons with identical G+C content.

Codons (without start and stop codons) are separated into four groups according to their number of G and C nucleotides and Spearman correlations with tRNA abundances are calculated separately for each of the four groups of codons. The number of codons in each group is indicated above each panel in parenthesis.

Figure S4

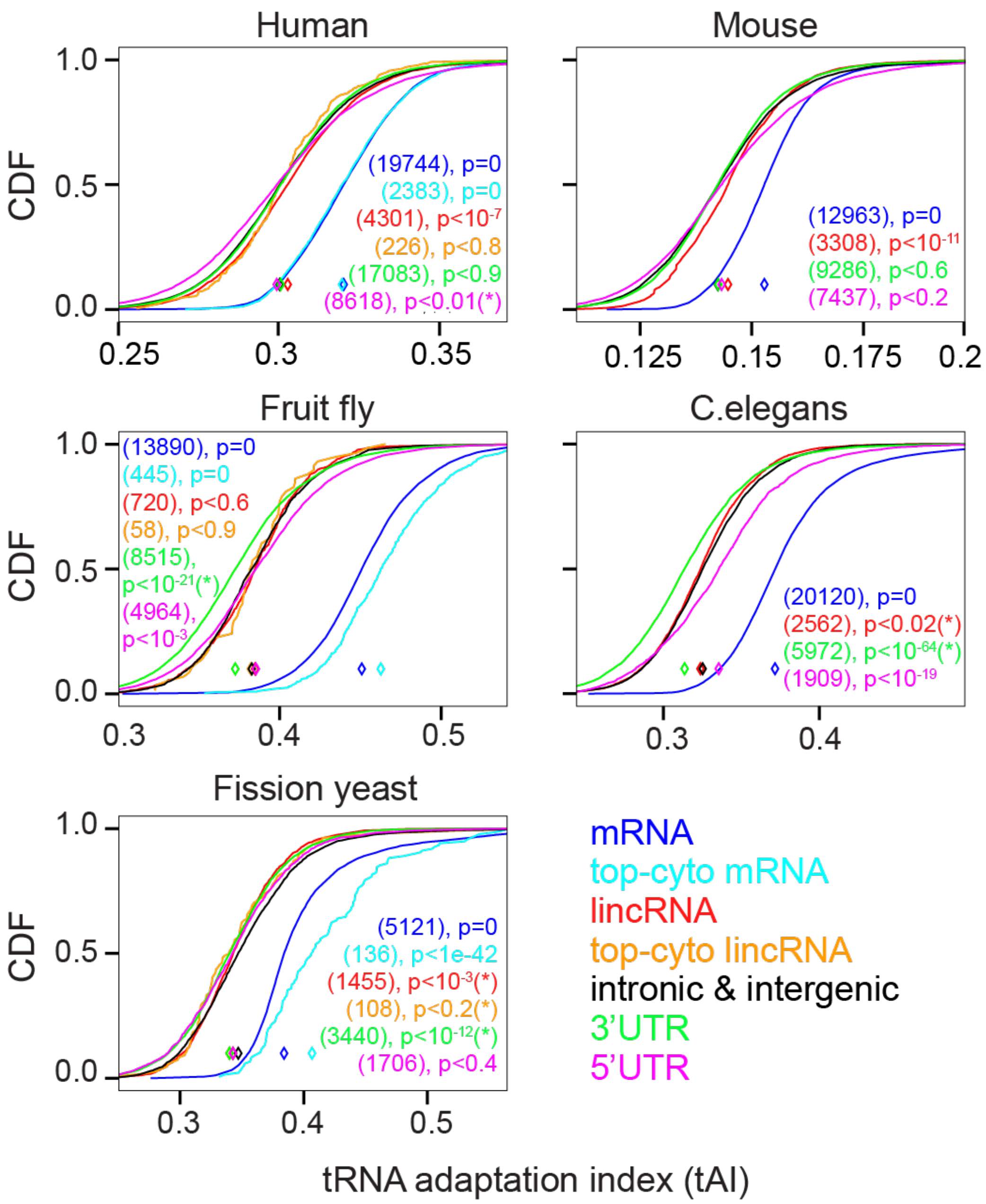

Figure S4: Cumulative distributions of tRNA adaptation indexes (tAIs) for different RNA types and five species.

tRNA adaptation index (tAI), calculated using tRNA gene counts (see Methods), for mRNA coding regions (blue), and longest ORFs in lincRNAs (red), intronic and intergenic control sequences (black), 3'UTRs (green) and 5'UTRs (magenta) in different species (see label above each panel). In the case of human, fruit fly, and fission yeast, tAIs of top-cytoplasmic mRNAs (light blue) and cytoplasmic lincRNAs (orange) are shown in addition. P values are indicated from Wilcoxon's rank-sum test comparing tAIs of control ORFs with those of ORFs in other RNA types (color-coded). P values are marked with an asterisk if the median tAI is below the one of control ORFs.

Figure S5

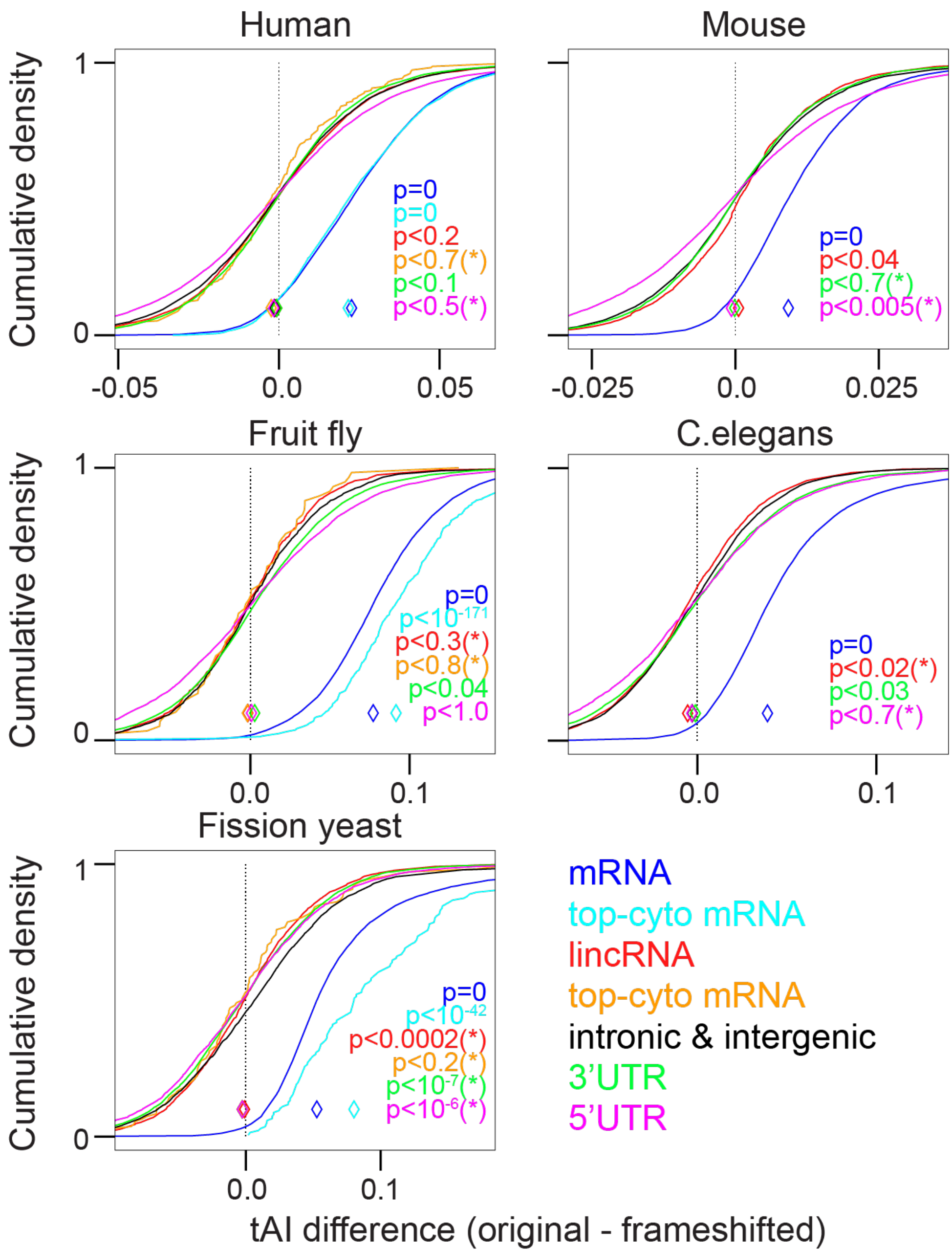

Figure S5: Comparison of tAIs between original and frame-shifted ORFs in five species.

Cumulative distributions of tAI differences between original and frameshifted ORFs for five species (indicated on top of each panel). tAI differences were calculated for mRNA coding regions (blue), and longest ORFs in lincRNAs (red), in intronic and intergenic control sequences (black), 3'UTRs (green) and 5'UTRs (magenta). For human, fruit fly, and fission yeast, tAI differences of top cytoplasmic mRNAs (light blue) and cytoplasmic lincRNAs (orange) are shown in addition. P values were calculated using Wilcoxon's rank sum test to compare the distribution of tAI differences for control sequences with those of other RNA types (color-coded). P values are marked with asterisks if the median tAI difference of that set of ORFs is below that of control ORFs.

Figure S6

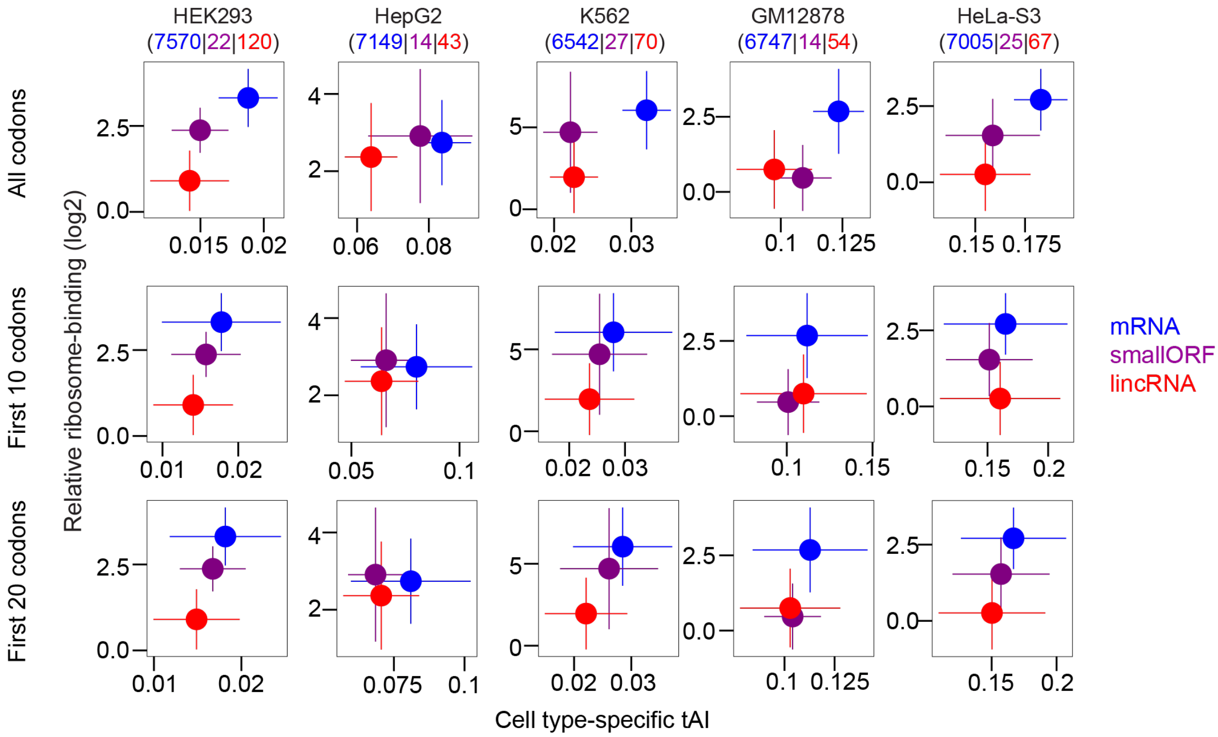

Figure S6: Correspondence between tAI and relative ribosome-binding in five human cell lines.

Correspondence between cell type-specific tAIs (x-axes) and relative ribosome-binding (y-axes) for three types of cytoplasmic RNAs, mRNAs (blue), annotated lincRNAs with small protein-encoding ORFs (smORFs; purple) and lincRNAs (red). tAIs are shown for all codons (first row), the first 10 codons (second row), and the first 20 codons (last row) after start codons, for five human cell lines (indicated on top of each column). Dots show the median values and error bars the median absolute deviations. Included are RNAs that are classified as cytoplasmic and have Ribo-Seq reads mapped to their longest ORF. The number of included RNAs of each type is indicated in parentheses at the top of each column with the RNA type color-coded.

There is a good correspondence between cell type-specific tAIs for the first (10 or 20) ORF codons and relative ribosome-binding for all cell lines, except where relative ribosome-binding is highly similar between RNA types, such as in HepG2 and for smallORFs and lincRNAs in GM12878.
